# Autonomous Stimulation of Cancer Cell Plasticity by the Human NKG2D Lymphocyte Receptor Coexpressed with Its Ligands on Cancer Cells

**DOI:** 10.1371/journal.pone.0108942

**Published:** 2014-10-07

**Authors:** Xin Cai, Zhenpeng Dai, Rebecca S. Reeves, Andrea Caballero-Benitez, Kate L. Duran, Jeffrey J. Delrow, Peggy L. Porter, Thomas Spies, Veronika Groh

**Affiliations:** 1 Clinical Research Division, Fred Hutchinson Cancer Research Center, Seattle, Washington, United States of America; 2 Human Biology Division, Fred Hutchinson Cancer Research Center, Seattle, Washington, United States of America; INSERM- CNRS- Univ. Méditerranée, France

## Abstract

The stimulatory NKG2D receptor on lymphocytes promotes tumor immune surveillance by targeting ligands selectively induced on cancer cells. Progressing tumors counteract by employing tactics to disable lymphocyte NKG2D. This negative dynamic is escalated as some human cancer cells co-opt expression of NKG2D, thereby complementing the presence of its ligands for autonomous stimulation of oncogenic signaling. Clinical association data imply relationships between cancer cell NKG2D and metastatic disease. Here we show that NKG2D promotes cancer cell plasticity by induction of phenotypic, molecular, and functional signatures diagnostic of the epithelial–mesenchymal transition, and of stem-like traits *via* induction of Sox9, a key transcriptional regulator of breast stem cell maintenance. These findings obtained with model breast tumor lines and xenotransplants were recapitulated by *ex* *vivo* cancer cells from primary invasive breast carcinomas. Thus, NKG2D may have the capacity to drive high malignancy traits underlying metastatic disease.

## Introduction

Cancer cell plasticity entails the development of traits enabling cancer cells to dissociate from primary tumor, disseminate, and expand clonally at distant sites. This process is regulated by the epithelial–mesenchymal transition (EMT) and the interrelated acquisition of regenerative cancer stem cell (CSC) attributes [1], [2]. Known drivers of cancer cell plasticity include heterotypic cues from tumor-associated stromal and/or immune system cells [1]. We previously identified an unconventional homotypic receptor–ligand interaction on cancer cells [3] and show here that resultant signaling induces reprogramming towards migratory and stem-like capacities.

The receptor involved, NKG2D (natural killer group 2 member D), is an activating lymphocyte receptor mainly on NK cells and CD8 T cells and is best known for mediating immune surveillance of virally infected and malignant cells [4]. Human NKG2D signals *via* the DAP10 (DNAX-activating protein 10) adaptor, which binds either PI3K (phosphoinositide 3-kinase) or Grb2 (growth factor receptor-bound protein 2), thus activating PKB/AKT (protein kinase B) or MAP (mitogen-activated protein) kinase cascades [5]. Ligands for NKG2D in humans include MICA and MICB (MHC class I-related chains A and B) and six members of the ULBP (UL-16 binding protein) family [6]. NKG2D ligands are largely absent from the surface of normal cells but can be induced by oncogenesis-associated stress responses in cancer cells [7]. This selective ligand expression enables NK cells and CD8 T cells to target cancer cells, at least at early tumor stages before immunosuppressive tactics of progressing tumors stifle this arm of the immune response [4], [8].

In addition to counteracting immune responses, some cancer cells co-opt NKG2D for their own benefit, complementing the presence of its ligands for self-stimulation of tumorigenesis [3]. Variable proportions of breast, ovarian, prostate, and colon cancer cells express signaling proficient NKG2D–DAP10 complexes, which activate the PI3K-AKT-mTOR (mammalian target of rapamycin) signaling axis and downstream effectors. Moreover, as in lymphocytes, NKG2D–DAP10 stimulates phosphorylation of ERK (extracellular signal-regulated kinase) and JNK in MAP kinase cascades [3]. Pathophysiological significance of NKG2D–DAP10 signaling is supported by a clinical association study that established positive correlations between percentages of cancer cells with surface NKG2D and tumor size and spread [3]. These relationships were extended by significant associations with lymph node metastasis, and by trend correlations with grade and lymphovascular invasion, suggesting NKG2D–DAP10 effects in tumor cell dissemination and metastasis formation [3]. The present study addresses the capacity of NKG2D–DAP10 to promote cancer cell plasticity underlying metastatic disease.

## Results

### Induction of EMT reprogramming by ligand stimulation of NKG2D

**Figure 1:**
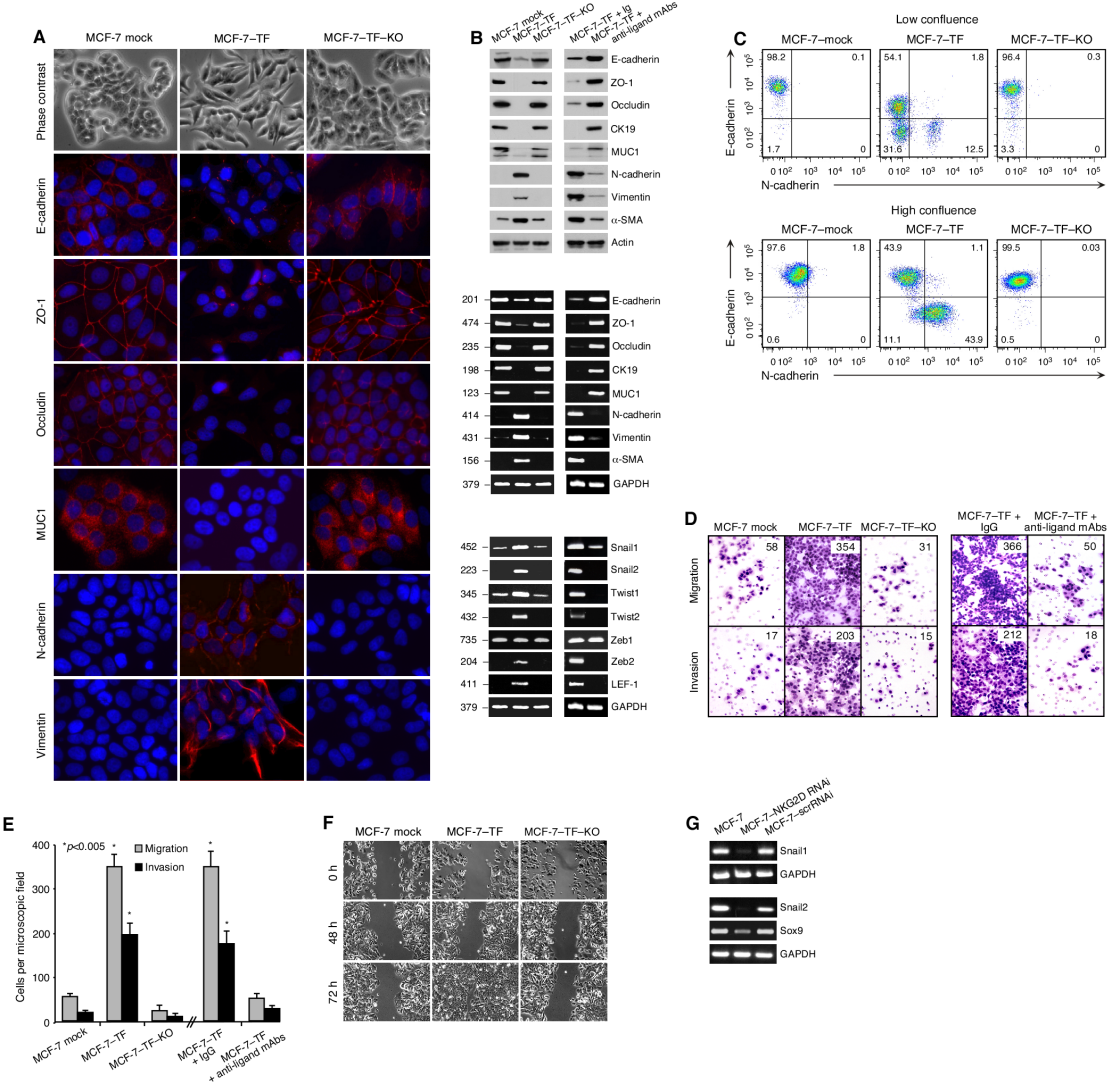
Induction of EMT-associated changes by NKG2D–DAP10 in transfected MCF-7–TF cells. (**A**) Phase contrast microscopy shows transition from epithelial to fibroblastoid shapes of MCF-7–TF versus mock-transfected control, and phenotypic reversion by MCF-7–TF–KO cells expressing NKG2D RNAi. By immunofluorescence microscopy, MCF-7–TF cells display diminished E-cadherin, ZO-1, occludin, and MUC1, and induced N-cadherin and vimentin. (**B**) Corresponding immunoblot (top panel) and RT-PCR (middle panel) data including the additional CK19 and α-SMA markers. Loss of epithelial and gain of mesenchymal markers is attenuated when MCF-7–TF cells are preincubated with antibody cocktail to NKG2D ligands. Bottom panel shows EMT-associated transcription factor RT-PCR profiles of MCF-7–TF and control lines and attenuation of loss and gain events by antibody masking of NKG2D ligands. (**C**) Flow cytometry of MCF-7-derived lines grown to low or high confluence for E-cadherin and N-cadherin. Numbers in quadrants indicate cell proportions in percent. Note that this detection is more sensitive then the procedure used in (**A**). (**D**) Exemplary data showing increased *in* *vitro* migration and invasion by MCF-7–TF versus mock transfected negative control and MCF-7–TF–KO cells. Presence of anti-ligand antibody cocktail reverses motility gains. Numbers indicate cell counts in randomly selected microscopic fields. (**E**) Graphic display of motility data with bars representing mean cell numbers (+/− SD) derived from three independent experiments with each four microscopic field counts. Asterisks denote *p*<0.005. (**F**) Imaging of MCF-7–TF cell migration versus control lines in wound-healing assays by phase contrast microscopy over time. (G) RT-PCR of Snail1, Snail 2, and Sox9 from parental, NKG2D-depleted or scrRNAi-transduced MCF-7 cells.

**Figure 2:**
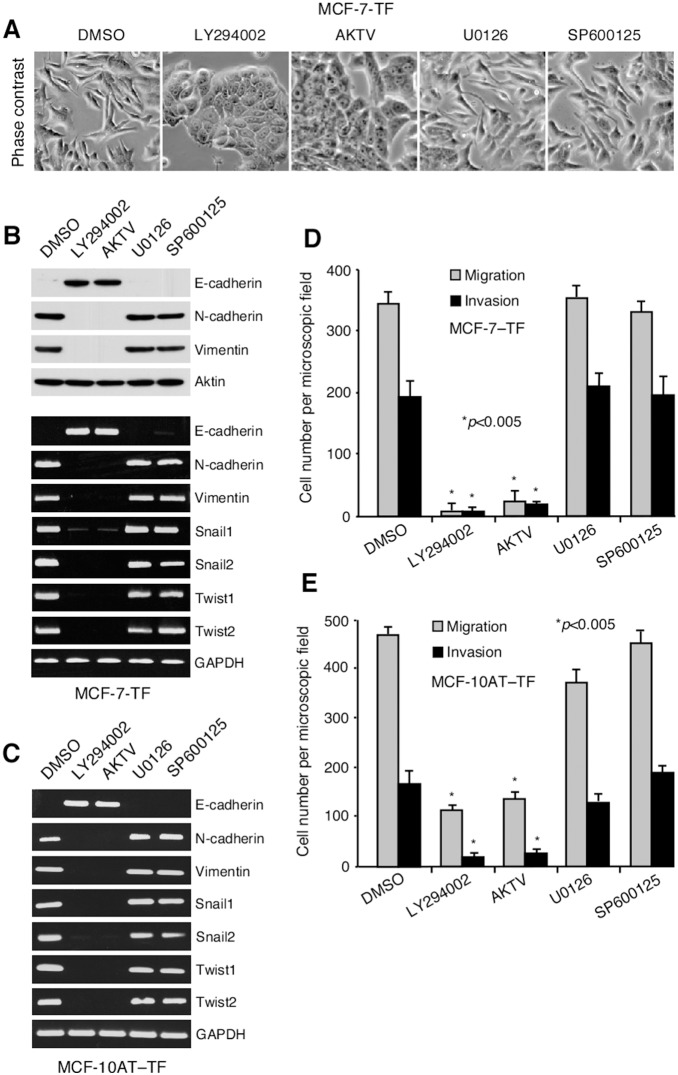
Dependence of EMT reprogramming on activation of PI3K-AKT. (**A**) By Phase contrast microscopy, MCF-7–TF cells revert to epithelial morphology when exposed to inhibitors of PI3K (LY294002) or pan-AKT (AKTV) but not of MEK1/2 (U0126) or JNK (SP600125). (**B**, **C**) Inhibition of PI3K or AKT reverses EMT protein marker and transcription factor profiles in MCF-7–TF and Dox-induced MCF-10AT–TF cells, shown by immunoblot and/or RT-PCR. (**D, E**) Inhibition of PI3K or AKT reduces migratory and invasive activities of MCF-7–TF and Dox-induced MCF-10AT–TF cells. Bars represent mean cell numbers (+/− SD) derived from three independent *in* *vitro* migration and invasion experiments with each four microscopic field counts. Asterisks denote *p*<0.005.

Epithelial tumor lines typically express NKG2D ligands but are either negative for their NKG2D–DAP10 receptor or, as with the MCF-7, BT-20, and MDA-MB-453 breast cancer lines, scarcely positive as reflected by minimal shifts of flow cytometry profiles and very low NKG2D and DAP10 mRNA and protein expression (Figure S1A and S1B; also refer to Figure 2B and Figure 2C in references 3 and 9, respectively). This paucity of the receptor in tumor lines is opposed to relative abundance, both by mRNA and protein expression, on positive *ex* *vivo* cancer cells (Figure S1C **–**E; also refer to Figure 1C–E in reference 3). To test in an *in* *vitro* model whether NKG2D induces EMT, we thus examined MCF-7 cells that were stably transfected with NKG2D–DAP10 (MCF-7–TF cells) resulting in surface expression at levels similar to *ex* *vivo* cancer cells (compare Figure S1C and Figure S1F). By phase contrast microscopy, MCF-7–TF cells displayed morphological transformations in comparison to mock-transfected control cells, which exhibited tightly clustered cobblestone-like shapes. MCF-7–TF cells, in contrast, displayed fibroblast-like morphologies (Figure 1A). These changes were due to ectopic expression of NKG2D–DAP10 as the parental phenotype was restored by recombinant lentivirus-mediated RNAi targeting of NKG2D in MCF-7–TF–KO cells (Figure 1A; for flow cytometry profiles of these model lines see Figure S1A and S1F). These observations suggested that ligand-mediated stimulation of NKG2D resulted in induction of EMT, which involves coordinated molecular and cellular changes leading to loss of epithelial cell-cell adhesion, polarity, and cytoskeletal integrity, concomitant with acquisition of mesenchymal protein signatures, spindle-cell shapes, and invasive and migratory abilities [10], [11]. Diagnostic of EMT are reduced expression of the cell junction-associated E-cadherin, and induction of N-cadherin and the cytoskeletal intermediate filament Vimentin. By immunofluorescence microscopy, MCF-7–TF cells displayed those changes in epithelial and mesenchymal marker proteins, which were reversed by RNAi targeting of NKG2D (Figure 1A). Corresponding results were obtained by immunoblot and RT-PCR profiling (Figure 1B). Extension of data to additional marker proteins and their corresponding mRNAs, including the epithelial tight junctional zona occludens-1 (ZO-1) and occludin, cytokeratin 19 (CK19) and mucin 1 (MUC1), and fibroblastoid α-smooth muscle actin (α-SMA), yielded conforming results (Figure 1A and 1B) [12]. EMT is orchestrated by transcription factors that include Snail1/2, Twist1/2, Zeb1/2, and LEF-1 [1]. Induction of the mRNAs for all of those factors except for Zeb1 was recorded with MCF-7–TF but not MCF-7–TF–KO cells (Figure 1B). Preincubation of MCF-7–TF cells with a cocktail of antibodies to relevant NKG2D ligands inhibited all recorded protein and mRNA changes, thus confirming ligand involvement (Figure 1B) [3]. Our previous study established the requirement of cell-cell contact for ligand-mediated stimulation of NKG2D–DAP10 signaling in cancer cells [3]. Accordingly, as seen by flow cytometry, the proportions of surface E-cadherin^−^/N-cadherin^+^ mesenchymal MCF-7–TF cells correlated with cell culture confluency (Figure 1C).

EMT-associated remodeling of cell-cell and cell-matrix adhesion endows cancer cells with migratory and invasive capabilities [10]. We tested for increased motility of MCF-7–TF cells in standard assays scoring migration through microporous membrane or traversion of reconstituted basement membrane (Matrigel). By comparison to the MCF-7 mock-transfected control, these experiments revealed about six- and 12-fold increases in migratory and invasive activities, respectively, of MCF-7–TF cells, which were suppressed in the NKG2D-depleted MCF-7–TF–KO cells (Figure 1D and 1E). For an alternative approach, scrape wound closure by confluent monolayers of MCF-7–TF and control cells was imaged by phase contrast microscopy. Whereas MCF-7–TF cells achieved complete wound closure within 72 h, little if any change was seen with the negative control cells (Figure 1F). Altogether, these results completed the *in* *vitro* studies of MCF-7–TF cells suggesting that NKG2D–DAP10 has the capacity to activate cancer cell EMT.

MCF-7 is a luminal breast cancer line with epithelial features as well as some constitutive epithelial to mesenchymal plasticity, which is typical of most breast tumor lines [13], [14]. Hence, Snail1 and Snail2 mRNAs were detectable by high-cycle (36 rounds) RT-PCR in untransfected MCF-7 parent cells (Figure 1G). Consistent with an involvement of endogenous NKG2D in Snail1/2 induction, NKG2D depletion in MCF-7–NKG2D RNAi cells led to reduction or loss of these transcripts as compared to scrambled RNAi control transductants (MCF-7–scrRNAi cells; Figure 1G; for flow cytometry profiles of these model lines see Figure S1A and S1G). Endogenous NKG2D may thus in principle have a capacity to promote differentiation towards mesenchymal signatures but these effects may not penetrate due to its scarce expression. Accordingly, presence or lack of endogenous NKG2D expression in breast cancer lines, such as the epithelial MCF-7 (NKG2D^+^) or mesenchymal SUM149PT (NKG2D^–^), is unaligned with their mainly epithelial versus mesenchymal representations [3], [9], [14], [15].

### Induction of cancer cell EMT is a general capacity of NKG2D–DAP10

For confirmation of principal findings across diverse tumor lines, we tested NKG2D–DAP10 transfectants of SUM149PT breast cancer [9], A375 melanoma [3], and MDAH-2774 ovarian cancer cells, and, in more detail MCF-10AT premalignant mammary epithelial cells, which were co-transduced with lentiviral constructs for doxycycline (Dox)-inducible NKG2D and DAP10 expression (Figure S2A and S2B). Distinct from MCF-7 cells, all of these four lines lack endogenous NKG2D (Figure S2A and S2B) [3], [9] but share the capacity to undergo EMT [13], [16]–[19]. As with MCF-7–TF cells, signaling proficiency of ectopically expressed NKG2D–DAP10 has been shown for the SUM149PT–TF and A375–TF lines [3], [9], and was confirmed with the newly generated MDAH-2774–TF and MCF-10AT–TF cells by immunoblot detection of phosphokinases *P*-Akt^S473^ and *P*-ERK1/2^T202/Y204^ after anti-NKG2D antibody crosslinking (Figure S2C).

With all four tumor lines, ectopic NKG2D–DAP10 expression (constitutive or Dox-induced) imprinted the morphological (shown only for MCF-10AT–TF cells), marker protein, and transcriptional EMT signatures recorded with MCF-7–TF cells (Figure S3A–D). Moreover, flow cytometry of Dox-induced MCF-10AT–TF cells for surface E-cadherin and N-cadherin confirmed that activation of EMT was cell contact- and hence presumably ligand-dependent (Figure S3E). All tumor lines with constitutive or Dox-induced ectopic NKG2D–DAP10 expression exhibited markedly increased *in* *vitro* migratory and invasive activities (Figure S3F).

### Dependence of EMT reprogramming on activation of PI3K–AKT

**Figure 3:**
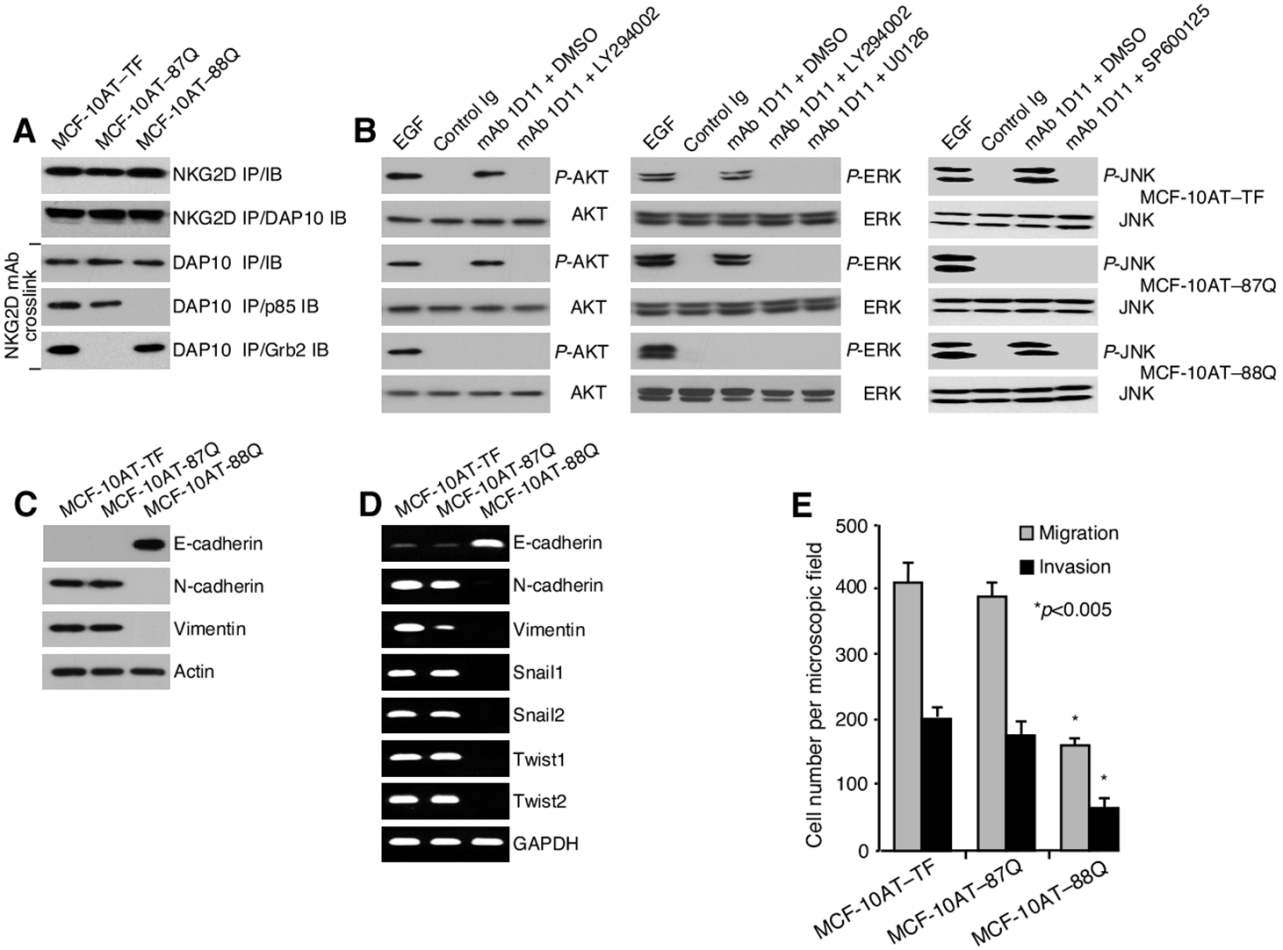
Dependence of EMT reprogramming on recruitment of PI3K/p85 but not Grb2. (**A**) Immunoprecipitation (IP) and immunoblot (IB) of NKG2D and/or DAP10 (N87Q* or M88Q*) in transduced MCF-10AT cells (top two panels). Bottom three panels show primary detection of DAP10 and the mutually exclusive association of its N87Q* and M88Q* variants with p85 and Grb2, respectively. (**B**) Signaling functionality of the DAP10 N87Q* and M88Q* variants. Note that ERK is downstream of PI3K. EGF was added for control activation, DMSO for solvent control. (**C**, **D**) Reversal of EMT protein marker and transcription marker profiles by expression of DAP10 (M88Q*) with impaired binding of p85, shown by immunoblot and/or RT-PCR. (**E**) DAP10 variant M88Q* but not N87Q* reduces migratory and invasive activities of Dox-induced MCF-10AT cells expressing the respective NKG2D–DAP10 mutant complexes. Bars represent mean cell numbers (+/− SD) derived from three independent *in vitro* migration and invasion experiments with each four microscopic field counts. Asterisks denote *p*<0.005.

To pinpoint NKG2D–DAP10 signaling requirements for EMT activation, MCF-7–TF and Dox-induced MCF-10AT–TF cells were exposed to pharmacological inhibitors of PI3K (LY294002), pan-AKT (AKT Inhibitor V), MAP kinase kinase (MEK1/2) upstream of ERK (U0126), and JNK (SP600125). Induction of all earlier examined EMT criteria including morphological changes (shown only for MCF-7–TF cells), diagnostic protein marker and transcription factor signatures, and motility, were dependent on the PI3K-AKT signaling axis with no discernible contribution of ERK and JNK (Figure 2A–E). In a complementary approach, NKG2D was expressed in MCF-10AT cells by transduction together with DAP10 mutated at either its PI3K/p85 (M88Q*) or Grb2 (N87Q*) binding site [20]. After confirmation of proper expression and function of the variant NKG2D–DAP10 complexes (Figure 3A and 3B), testing for induction of EMT parameters ascertained their dependence on recruitment of PI3K/p85 but not of Grb2 (Figure 3C–E).

### Association of NKG2D–DAP10 with EMT signatures of *ex vivo* cancer cells

**Figure 4:**
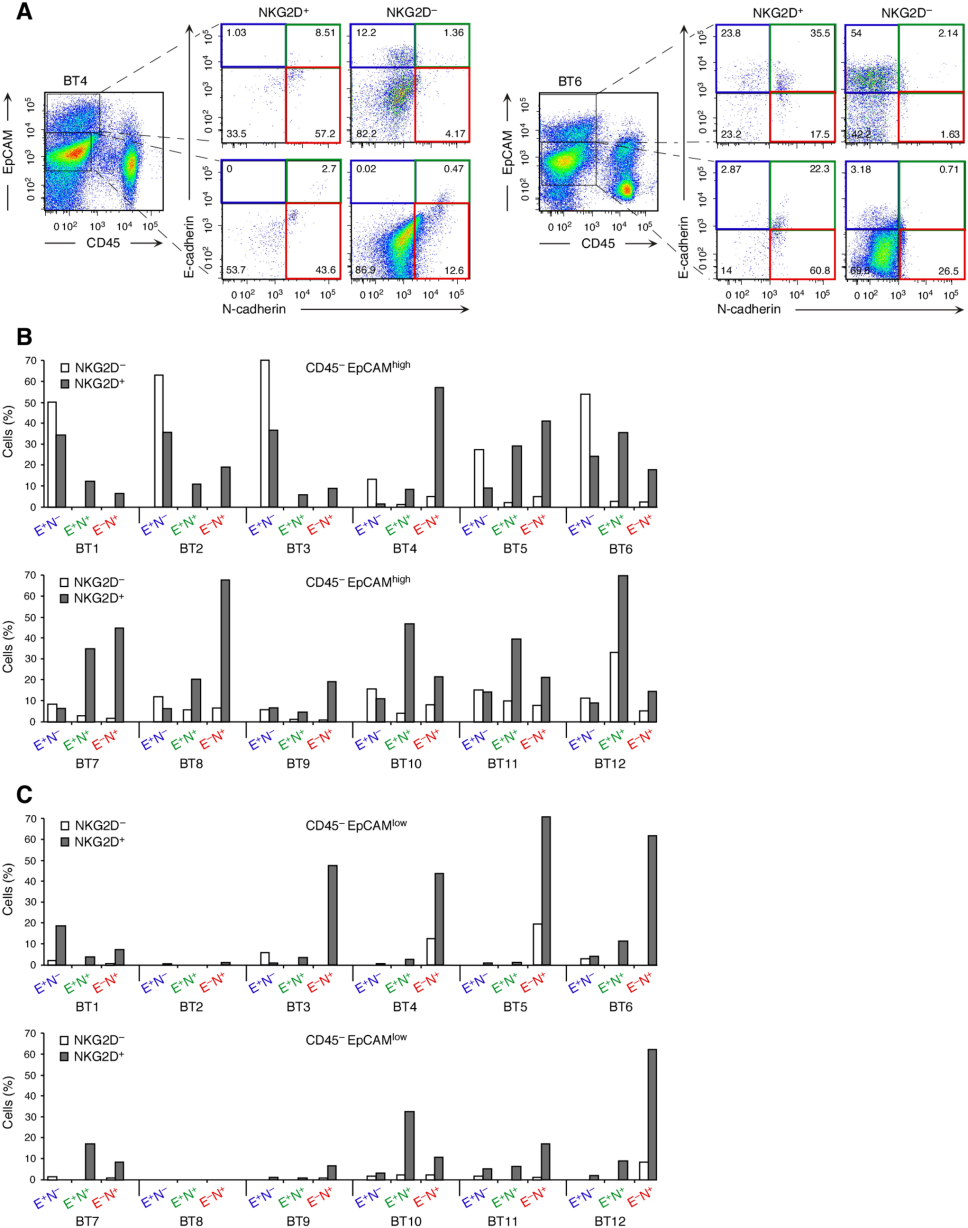
Association of NKG2D with hybrid and mesenchymal E-cadherin/N-cadherin signatures on *ex vivo* breast cancer cells and diminished expression of NKG2D ligands on EMT-transitioned cancer cells. **(A)** Examples (breast cancer specimens BT4 and BT5) of multiparameter flow cytometry dot plots illustrating cancer cell gating based on absence of CD45 and presence of EpCAM (high or low) and subsequent display of E-Cadherin/N-cadherin phenotypes of NKG2D^+^ or NKG2D^–^ cancer cell subsets. Numbers in quadrants indicate cell proportions in percent. Quadrant gates are based on fluorescence-minus-one isotype Ig control stainings. **(B, C)** Graphic display of data derived from 12 breast cancer specimens (BT1–BT12) that were analyzed as in **(A)**. E^+^N^–^, E^+^N^+^, and E^–^N^+^ refer to E-cadherin and N-cadherin status. Colors correspond to dot plot quadrants in **(A)**. See main text for further explanation.

Signaling proficiency of the NKG2D–DAP10 receptor in breast cancer cells has been documented [3] and was confirmed here with two additional breast cancer specimens (Figure S1H). Antibody-mediated receptor crosslinking induced AKT phosphorylation downstream of PI3K in breast cancer cells sorted for absence of CD45 (hematopoietic cell exclusion), expression of EpCAM (cancer cell inclusion), and NKG2D. Appearance of phospho-AKT (*P*-AKT) was sensitive to Ly294002 and thus dependent on PI3K. To ascertain biological relevance of NKG2D–DAP10 in EMT reprogramming, freshly isolated cell suspensions from 12 primary invasive breast cancer specimens (referred to as BT1 to BT12) were examined by multi-parameter surface flow cytometry for CD45, EpCAM, NKG2D, and the E-cadherin^−^/N-cadherin^+^ EMT signature. EpCAM is a *bona fide* cancer cell marker although downregulation may occur during EMT [12], [21]. Indeed, EpCAM expression among CD45^–^ cell populations was heterogeneous in all but one (BT8) of the 12 tumor cell suspensions (Figure 4A). We thus examined CD45^–^EpCAM^high^ and CD45^–^EpCAM^low^ cells separately for surface NKG2D and E-cadherin/N-cadherin patterns. Consistent with our earlier findings [3], NKG2D^+^ cancer cells were present in all 12 breast cancer specimens, ranging between 0.5 and 27.8% (mean 9.7 +/− 9.1%) of total CD45^–^EpCAM^high^ cells (Table S1). EMT represents a continuum with epithelial/mesenchymal hybrid and fully transitioned mesenchymal-like states [10], [11], [21]. Based on our findings with the model tumor lines, cancer cell NKG2D should thus be associated with both hybrid (E-cadherin^+^N-cadherin^+^) and further differentiated (E-cadherin^–^N-cadherin^+^) phenotypes. In 9 of the 12 tumor cell suspensions tested (BT4 to BT12), most NKG2D^+^ among the CD45^–^EpCAM^high^ cells had E-cadherin/N-cadherin patterns consistent with either partial (E-cadherin^+^N-cadherin^+^) or more progressed (E-cadherin^–^N-cadherin^+^) EMT (Figure 4A and 4B). Except for the BT8, BT11, and BT12 suspensions, skewing towards those phenotypes was not apparent among the matched NKG2D^–^ cancer cells. In three cases (BT1, 2, and 3), cancer cells with mixed or mesenchymal signatures were confined to the NKG2D^+^ subset but were not predominant (Figure 4B).

NKG2D^+^ cells were also noted among CD45^–^EpCAM^low^ tumor cell populations, comprising between 0.3 and 59.3% (mean 12.6 +/− 18.9%) (Table S1). In all but the BT1 sample, NKG2D^+^CD45^–^EpCAM^low^ populations also preferentially displayed EMT phenotypes. Notably, in select samples (BT3, BT5, BT6, and BT12), proportions of mesenchymal (E-cadherin^–^N-cadherin^+^) cells were considerably larger among NKG2D^+^CD45^–^EpCAM^low^ compared to NKG2D^+^CD45^–^EpCAM^high^ cells, suggesting that EpCAM downregulation may occur late in the EMT process (Figure 4B and 4C). Altogether, these observations corroborated the findings made with the model tumor lines, supporting a prominent role of NKG2D as a natural activator of EMT in cancer. This is consistent with the fact that the NKG2D^+^ cells constituted substantive proportions when recorded as percent of total hybrid and mesenchymal marker profile-positive cells (Table S1). All 12 tumor cell suspensions contained varying proportions of CD45^–^EpCAM^–^ cells that lacked E-cadherin and N-cadherin and thus could not be assigned to a specific cell type. With the exception of four tumor specimens (BT2, BT3, BT11, and BT12), NKG2D^+^ cells were absent among those populations.

### NKG2D induces EMT in a tumor xenotransplant model

**Figure 5:**
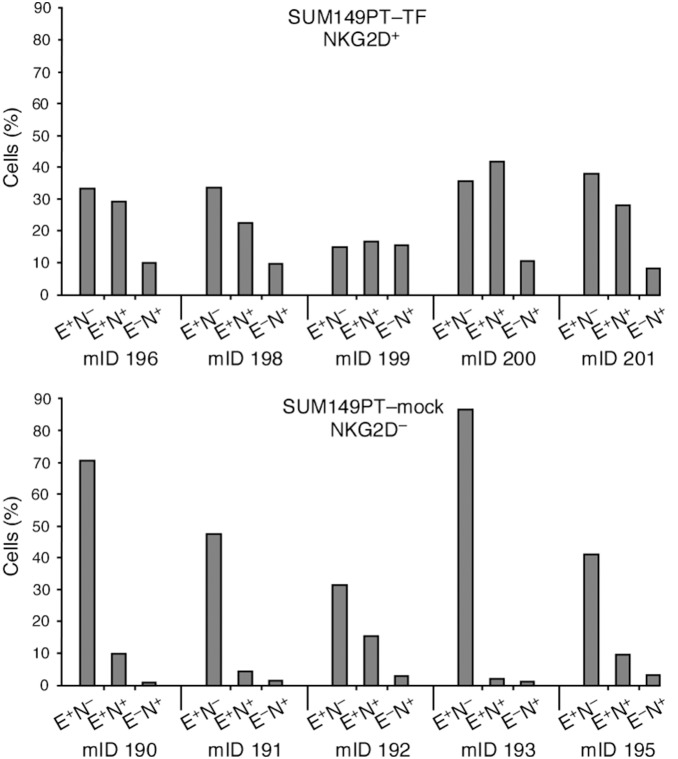
Association of NKG2D with mesenchymal and hybrid EMT phenotypes in orthotopic xenograft-derived tumors. Cell suspensions from tumors derived from immunodeficient mice orthotopically xenografted with breast cancer SUM149PT–TF cells (upper bar graph) are strongly skewed towards hybrid E-cadherin^+^/N-cadherin^+^ and further transitioned E-cadherin^−^/N-cadherin^+^ phenotypes in comparison to control mock-transfected SUM149PT cell tumors (lower bar graph). E^+^N^–^, E^+^N^+^, and E^–^N^+^ refer to E-cadherin and N-cadherin status; mID, mouse identification number.

Complementary evidence for a role of NKG2D in EMT was obtained from tumors derived from SUM149PT–TF or negative control cells orthotopically xenografted into mammary fat pads of NOD/SCID mice, a model experiment that was instrumental in establishing NKG2D-driven tumorigenicity [9]. More direct *in vivo* evidence was not attainable since spontaneous or carcinogen-induced cancers in mice lack NKG2D expression [3]. The SUM149PT line is phenotypically heterogeneous, harboring sizeable subsets of cells with mesenchymal marker profiles [14]. Hence, immunohistochemistry may pose difficulties in assessments of EMT-associated changes. Examination of xenograft tumor-derived cell suspensions by flow cytometry exposed strongly increased representations of hybrid/mesenchymal phenotypes in all of five NKG2D^+^ SUM149PT–TF as opposed to NKG2D^–^ mock control tumors (Figure 5). EMT-associated migratory activity of xenotransplanted SUM149PT–TF cells may have contributed to enhanced tumor cell dissemination previously observed in this animal model [9].

### Instruction of stemness reprogramming by NKG2D

**Figure 6:**
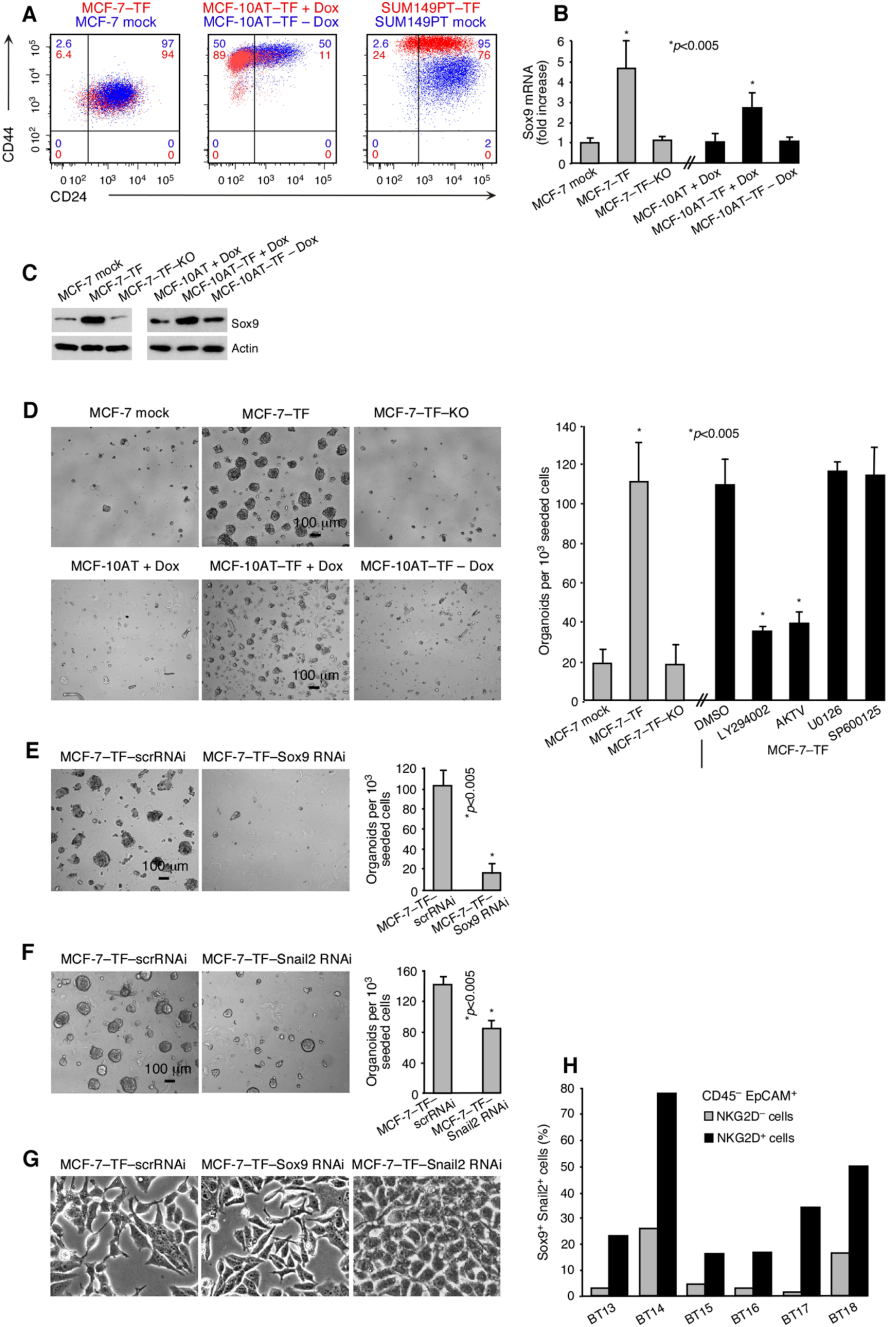
Association of NKG2D with breast cancer stem-like attributes. (**A**) Two-color flow cytometry of CD24/CD44 patterns in stably transfected MCF-7–TF and SUM149PT–TF, and Dox-induced transduced MCF-10AT–TF cells (all shown in red) compared to negative controls (shown in blue). Colored numbers in quadrants indicate corresponding cell proportions in percent. Data shown are representative of three experiments. (**B**) Quantitative RT-PCR of Sox9 in the MCF-7 and MCF-10AT-derived model lines. Experimental data bars represent fold-increases over negative control data set at one. Data are representative of three independent experiments. Asterisks denote *p*<0.005. (**C**) Corresponding immunoblot data for Sox9 and actin control. (**D**) Phase contrast micrographs of mammospheres formed by MCF-7–TF and induced MCF-10AT–TF versus negative control lines. Bar graph at right (grey bars) summarizes data as number of organoids (>100 µm diameter) counted in three sets of triplicate wells each seeded with 10^3^ MCF-7–TF cells. Black bars represent experiments performed with MCF-7–TF cells under the same conditions in the presence of inhibitors of PI3K (LY294002), pan-AKT (AKTV), MEK1/2 (U0126), or JNK (SP600125). Data are representative of three independent experiments. DMSO serves as solvent control. (**E**, **F**) As in (**D**), with MCF-7–TF cells expressing Sox9 or Snail2 RNAi or scrRNAi controls. (**G**) Phase contrast micrographs of mesenchymal versus epithelial cell morphologies of cell lines presented in (**E**, **F**). (**H**) Graphic display of proportions of Sox9^+^/Snail2^+^ cells among NKG2D positive or negative CD45^–^EpCAM^+^ *ex vivo* breast cancer cells (specimens BT13 to BT18) determined by multi color flow cytometry.

In addition to conferring migratory abilities, EMT transcription factors Snail1 and Twist regulate surface marker profiles that define breast cancer cell populations enriched for cells with stem-like attributes [22]–[24]. Consistent with the induction of Snail1 and Twist, ectopic NKG2D–DAP10 expression (constitutive or Dox-induced) was associated with breast cancer stem cell-like CD24^−^/CD44^+^ profile shifts in the MCF-7–TF, MCF-10AT–TF and SUM149PT–TF lines (Figure 6A) [22], [25]. However, only a small fraction of cells with CSC-like phenotypes qualify as functional CSCs [2]. More relevant to EMT–stemness interrelations may be the fact that EMT transcription factors, such as Snail2 or Zeb1, in cooperation with separate genetic circuitries, also regulate functional breast CSC states [2], [26]–[28]. By microarray gene expression profiling, we identified a prominent induction of the Sox9 transcription factor in MCF-7–TF compared to mock-transfected control cells (see microarray data at http://www.ncbi.nlm.nih/geo/under accession code GSE53961), which was independently confirmed by quantitative RT-PCR and immunoblot (Figure 6B and 6C).

Sox9 acts cooperatively with Snail2 (Slug) and to a lesser extent Snail1 as a master regulator of the mammary stem cell state [28]. Thus, by positively regulating Sox9 in addition to Snail2 and Snail1 (see Figure 1B), NKG2D may have the capacity to promote functional stem cell-like attributes. This notion was supported by three-dimensional matrigel organoid cultures and classical mammosphere assays. In both types of experiments, MCF-7–TF cells formed numerous large (>100 µm) organoids whereas mock-transfected control or MCF-7–TF–KO cells generated only few cell clusters that were typically small (Figure 6D). As with EMT reprogramming, organoid formation was dependent on PI3K–AKT with no apparent involvement of ERK or JNK (Figure 6D). Corresponding evidence for the capacity of NKG2D to induce Sox9 and organoid formation was obtained with Dox-induced MCF10AT–TF cells (Figure 6B–D).

Sox9 and Snail2 are both required for mammary epithelial cells to enter and stably maintain a functional stem cell state [28]. Accordingly, RNAi-mediated depletion of Sox9, and separately of Snail2, abrogated organoid formation in MCF-7–TF mammosphere cultures [Figure 6E and 6F). Unlike Snail2, which is thought to contribute to stem cell induction *via* activation of an EMT, effects of Sox9 in EMT reprogramming are considered minor [28]. Consequently, EMT-associated morphological changes of MCF-7–TF cells were thus reversed by RNAi-mediated depletion of Snail2 but not of Sox9 (Figure 6G).

The defining hallmark of highest plasticity cancer cells is their efficient tumor initiation upon xenografting in immunodeficient mice. Our earlier mouse model study demonstrated markedly reduced latencies and enhanced tumor incidences with orthotopically transplanted MCF-7–TF versus control lines [9]. Those outcomes are consistent with NKG2D-mediated induction of Sox9 and Snail2 and attendant acquisition of CSC capacities. This induction also provided an explanation for the observed enhanced tumor initiation by untransfected MCF-7 cells expressing minimal endogenous NKG2D (Figure 1G; for flow cytometry profiles of the parental MCF-7, MCF-7–NKG2DRNAi, and MCF-7–scrRNAi lines see Figure S1A and S1G) [9]. To probe for relevance in human cancers, *ex vivo* tumor cells from six primary invasive breast cancer specimens (BT13 to BT18) were tested for associations between NKG2D and Sox9 and Snail2 by polychromatic flow cytometry. In all tumors, proportions of CD45^–^EpCAM^+^ cells co-expressing Sox9 and Snail2 were significantly larger among NKG2D^+^ compared to NKG2D^–^ cells (Figure 6H). Altogether, these results support tumorigenic significance of NKG2D *via* Sox9- and Snail2-mediated induction of CSC traits.

## Discussion

High plasticity cancer cells are considered main culprits of tumor dissemination and rebounding following conventional cancer therapy [2], [29]. The present study indicates that co-opted expression of, and signaling by, NKG2D on cancer cells promotes cancer cell plasticity with differentiation towards mesenchymal phenotypes and dissemination-enabling and tumor-initiating capacities. Physiological significance is supported by the recapitulation of EMT and Sox9 signatures recorded with model tumor lines by primary invasive breast cancers. It is likely that the role of NKG2D as a driver of cancer cell plasticity is broadly applicable, extending to at least ovarian, colon, and prostate carcinoma cells [3].

The relevance of EMT, and by inference of stemness reprogramming, in human tumor development is not uncontroversial [30]. However, recent flow cytometry-based demonstrations of circulating tumor cells with epithelial, mesenchymal, or hybrid signatures, and associated metastasis-initiating capacities, constitute direct evidence for human cancer cell plasticity and its pathophysiological significance [21], [31]. Our multi-parametric single-cell analysis of *ex vivo* tumor cell suspensions extends this evidence to primary breast cancers and uncovers that mixed epithelial/mesenchymal and mesenchymal-like breast cancer cell phenotypes are more prevalent and presumably more complex than previously thought. Because most NKG2D^+^ breast cancer cell populations examined were skewed towards hybrid and mesenchymal phenotypes and represented substantive proportions of all cells with those differentiation stages, NKG2D may quite possibly have a prominent role in EMT induction. A reverse scenario with NKG2D induction as a result of EMT is unlikely as considerable proportions of hybrid or mesenchymal cancer cells lacked NKG2D and varying subsets of NKG2D^+^ cancer cells retained epithelial marker profiles.

The NKG2D-mediated induction of Sox9 alongside Snail2 (and Snail1) is of particular interest as these factors have been functionally linked in mouse mammary epithelial and human breast CSC biology [28]. Both Sox9 and Snail2 are considered central for induction and maintenance of stem cell traits, with each factor contributing distinct attributes but both being required for stem cell function [28]. Our findings with the MCF-7–TF line and its Sox9- or Snail2-depleted variants are consistent with this functional dichotomy and confirm with human cells the necessity of both factors for induction of functional stem cell-like abilities. NKG2D-mediated activation of major oncogenic signaling pathways is compatible with its ability to induce Sox9 although specific intermediates are unidentified [32], [33].

EMT and the regulation of stem cell states are of broader significance due to their involvement in embryogenesis. NKG2D receptor expression in embryonic tissues has not been reported, however, and NKG2D-deficient mice have no overt developmental abnormalities. But at least in mice, some NKG2D ligands are expressed at distinct embryonic stages, mostly in the central nervous system, and are present on subventricular zone-derived neural stem/progenitor cells where they stimulate proliferation and survival [34], [35]. Hence, it remains possible that non-lymphocytic NKG2D expression and function during development may have gone unnoticed thus far.

Our results add a provocative twist to current knowledge as cancer cells may co-opt NKG2D as an oncoprotein serving their own benefit. Immune surveillance of advanced tumors is progressively thwarted by NKG2D ligand-mediated negative imprints on lymphocyte effector functions [8], [36]. The here described capacity of NKG2D to promote high malignancy traits strongly implies the possibility that the receptor itself may represent the main factor underlying the typically poor clinical outcomes that have been associated with cancer cell expression of its ligands [37]–[39].

In conclusion, the evidence presented here supports a major role of NKG2D in tumorigenesis *via* promotion of cancer cell plasticity. Various efforts targeting lymphocyte NKG2D or its ligands for cancer therapy are underway [40]–[43]. Those approaches are solely based on knowledge of its immune related functions and may thus be misdirected as underlying assumptions are incomplete.

## Materials and Methods

### Tumor lines, breast cancer specimens, and cancer cell suspensions

Tumor lines MCF-7, BT-20, MDA-MB-453, MDAH-2774, and A375 (American Type Culture Collection, ATCC) were grown in RPMI 1640/10% fetal bovine serum (FBS); MCF-10AT (Barbara Anne Karmanos Cancer Institute) in DMEM:F-12 (ATCC)/5% horse serum (Invitrogen)/EGF (20 ng/ml)/insulin (10 µg/ml)/hydrocortisone (0.5 µg/ml)/cholera toxin (100 ng/ml) (all from Sigma)/penicillin (100 units/ml)/streptomycin (100 µg/ml; Invitrogen); and SUM149PT (ATCC) in Ham’s F12 (Invitrogen)/5% FBS/hydrocortisone/insulin (5 µg/ml)/penicillin/streptomycin. Surgical specimens of primary invasive breast cancer and associated pathology annotations were obtained from the Cooperative Human Tissue Network (CHTN; www.chtn.nci.nih.gov) and the University of Washington/Fred Hutchinson Cancer Research Center (FHCRC) Cancer Consortium Breast Specimen Repository & Registry (BSRR) under FHCRC Institutional Review Board-approved Research Not Involving Human Subjects protocol #6007/552. All specimens have been deidentified prior to access. For pathology parameter annotations see Table S1. Fresh specimens were processed to single-cell suspensions using a Human Tumor Tissue Dissociation Kit and a gentleMACS Dissociator (both from Miltenyi Biotech). SUM149PT-derived xenograft tumors were harvested in strict accordance with the recommendations in the Guide for the Care and Use of Laboratory Animals of the National Institutes of Health and approved under the active FHCRC Institutional Animal and Use Committee (IACUC) protocol #1870. Xenograft tumor processing was performed as described [9].

### NKG2D–DAP10 expression, siRNA transduction, and RT-PCR

The MCF-7, SUM149PT, and A375-derived NKG2D–DAP10 transfectants and mock controls, and NKG2D RNAi and scrRNAi constructs and transduction have been described [3], [12]. MDAH-2774 transfectants were generated accordingly. Dox-inducible expression of NKG2D (*KLRK1*; GenBank accession number X54870), and DAP10 (*HCST*; GenBank accession number AF072844) and its N87Q* and M88Q* mutants, was directed by insertion of RT-PCR amplicons flanked by *Bam* HI and *Sma* I sites (underlined) into lentiviral pLVCT-tTR-KRAB constructs (plasmid 11643, Addgene). For detailed information and oligonucleotide primers see **Materials and Methods S1 in File S1**. MCF7–TF cells were transduced with commercial Sox9 or Snail2 constructs (pLKO.1-sh-hSOX9-1 and siSlug2, both from Addgene). RT-PCR primers for NKG2D and DAP10 have been described [3]. Primers for EMT-associated transcription factors and cellular markers and technical information are listed under **Materials and Methods S1 in File S1**.

### Gene expression microarrays

For RNA conversion and biotin-labeling see **Materials and Methods S2 in File S1**. Biotin-labeled cRNA was processed on a HumanRef8v3 Expression BeadChip (Illumina) and imaged using the Illumina iScan system. Microarray data were assessed for quality followed by quantile normalization using the Bioconductor package *lumi* [44]. The data set was initially filtered by flagging probes that fell below a signal noise floor, which was established using three standard deviations of the negative control probe signals within each array. Subsequent data set filtering employed a variance filter using the ‘shorth’ function of the Bioconductor package *genefilter*. Pair-wise statistical analyses were performed using the Bioconductor package *limma* [45]. Illumina BeadChips are constructed using probes design with a 3′-UTR bias. As such, the probe for *KLRK1* encoding NKG2D (chr12∶10525415–10525464) targets its untranslated region and therefore fails to bind to the 3′-UTR-truncated NKG2D cDNA expressed in the MCF-7–TF line.

### Immunoprecipitations, immunoblots, and pharmacological inhibitors

NKG2D and DAP10 were co-immunoprecipitated from NP-40 buffer lysates of sorted CD45^–^EpCAM^+^ breast cancer cells, and of MCF10AT–TF and MDAH-2774–TF and control cells using mAb 5C6 immobilized on AminoLink Plus Coupling Resin (Pierce) [3]. With parental control cells, lysates were from 5×10^7^ cells [3]. Immunoblots were probed with polyclonal antibodies to NKG2D or DAP10 (N-20 and N-17, Santa Cruz Biotechnology) and developed using secondary reagents and Supersignal West Pico Chemiluminescent Substrate (Thermo Scientific). For *P*-AKT induction, sorted CD45^–^EpCAM^+^ breast cancer cells or tumor lines (2×10^6^ cells per experimental condition) were desensitized for 4–24 h in serum-free RPMI medium at 37°C before exposure in 0.5 ml RPMI to insulin (Sigma; 5 µg/ml, 35 min at 37°C), EGF (rhEGF, Sigma; 100 ng/ml, 10–20 min at 37°C), or purified mAb 1D11 (5 µg/ml, 30 min at 4°C). For crosslinking, washed cells in 0.2 ml RPMI were exposed to goat anti-mouse F(ab’)_2_ (Jackson ImmunoResearch; 20 µg/ml, 5 min at 37°C). Ly294002 (20 µM; Cell Signaling Technology) was applied 30 min before stimulations. Reactions were terminated on ice with 1 ml sodium orthovanadate (Na_3_VO_4_, Calbiochem; 2 mM in cold PBS) and cells resuspended in NP-40 lysis buffer with protease inhibitor cocktail (Roche) and Na_3_VO_4_. Cleared supernatants were subjected to SDS-PAGE (4–12% gradient NuPAGE gels, Invitrogen), and proteins electroblotted onto PVDF membranes (Immobilon-P, Millipore) and probed with rabbit anti-human *P*-AKT^S473^ (clone 193H12) or pan-AKT (clone C67E7), secondary HRP-conjugated anti-rabbit IgG (all from Cell Signaling Technology), and chemiluminescent reagent. *P*-ERK was detected with cells desensitized for 24 h. Ly294002 (20 µM) or an inhibitor of MEK/ERK (U0126, 10 µM; Cell Signaling Technology) were added 30 min before stimulations. Samples were processed as above and immunoblots probed using rabbit mAb to *P*-p44/42 MAPK (ERK1/2^T202/Y204^; clone 137F5) and polyclonal antibodies to protein controls (all from Cell Signaling Technology). Other inhibitors used were AKTV (20 µM; EMD Millipore) and SP600125 (100 µM; Sigma).

EMT markers were detected by probing total cell lysate immunoblots with antibodies to human E-cadherin (clone 36), N-cadherin (clone 32; both from BD Transduction Laboratories), occludin (clone Z-T22), ZO-1 (clone Z-R1; both from Invitrogen), vimentin (clone RV202, BD Pharmingen), α-SMA (clone 1A4, Sigma), CK14 (clone AF64, Covenance), CK18 (clone RGE53, Santa Cruz Biotechnology), CK19 (clone EP1580Y, Epitomics), and MUC1 (clone VU4H5, Cell Signaling Technology), followed by secondary HRP-conjugated anti-rabbit or anti-mouse IgG (Cell Signaling Technologies) and chemiluminescent reagent (Thermo Scientific). Sox9 was detected using rabbit polyclonal antibody (Abcam).

### Immunofluorescence microscopy

For immunocytochemistry of EMT markers, formaldehyde-fixed and permeabilized cells (5 min in 0.2% Triton X-100/PBS) grown on Nunc Lab-Tek II CC2 chamber slides (Fisher) were treated for 1 h with blocking buffer (5% goat serum in SuperBlock; Pierce) and sequentially stained in humid chambers with titrated concentrations of mAb (clones and sources as above) to E-cadherin, N-cadherin, occludin, ZO-1, Vimentin, or MUC1, and secondary antibody (goat anti-mouse IgG–Alexa Fluor 594; Invitrogen). Washed, nuclear counterstained (DAPI; Invitrogen), and coverslipped slides were examined using a Nikon Eclipse E800 microscope. Phase contrast images were taken with a DeltaVision microscope (Applied Precision).

### Flow cytometry

Cell lines and tumor cell suspensions in PBS/10% human serum/0.15% sodium azide were variably incubated with pre-titrated mAb-fluorochrome conjugates to NKG2D (clone 1D11; APC), CD45 (clone 2D1; APC-H7), CD24 (clone ML5; PE), CD44 (clone G44-26; PerCP-Cy5.5; all from BD Pharmingen), EpCAM (clone 9C4, Alexa Fluor 488), E-cadherin (clone 67A4; PerCP/Cy5.5), and N-cadherin (clone 8C11; PE; all from Biolegend). DAPI was used for live/dead cell distinction. Sox9 and Snail2 were tested in CD45/EpCAM (clone 9C4, PerCP/Cy5.5; Biolegend)/NKG2D surface labeled, fixed (Fixation Buffer) and permeabilized (Permeabilization/Wash Buffer; both from R&D Systems) cells using anti-SLUG mAb (clone 51772, FITC; Abcam) and anti-SOX9 mAb (clone 76997; Abcam) together with PE–goat anti-mouse IgG2a (Southern Biotech). Live/dead cell distinction was with LIVE/DEAD Fixable Aqua Dead Cell Stain Kit (Invitrogen). Isotype-specific Ig were used as controls and background fluorescence subtracted. Stained cells were analyzed using a BD LSRII flow cytometer (BD Biosciences) and FlowJo software (Tree Star).

### Tumor cell invasion/migration, matrigel organoid cultures, and mammosphere assays

BD BioCoat Matrigel Invasion Chambers (Discovery Labware) were seeded and cells cultured, harvested, and counted as described in **Materials and Methods S3 in File S1**. Migration assays were identical except that Control Inserts (Discovery Labware) were used instead of the Matrigel Invasion Chambers. Cell migration in wound-healing assays was imaged in regular intervals by phase contrast microscopy. Matrigel organoid cultures were as described [29]. For mammosphere assays, cells were cultured in 96-well ultra low attachment plates (1×10^3^ cells/well) in serum-free MammoCult medium (Stemcell Technologies) with 0.9% methylcellulose (Sigma Aldrich). After 7 days, mammospheres with >100 µm diameters were microscopically counted in three sets of triplicate wells.

### Statistical analyses

Numerical data are presented as mean +/− standard deviation. Student’s *t* test was used to calculate *p* values, with *p*<0.05 assigned significance.

### Accession Numbers

Microarray data are available at http://www.ncbi.nlm.nih.gov/geo/ under accession code GSE53961.

## Supporting Information

Figure S1 **NKG2D–DAP10 in breast tumor lines and** ***ex*** ***vivo*** **breast cancer cells.** (**A–C, F, G**) Flow cytometry profiles of surface NKG2D (blue lines) on *ex* *vivo* breast cancer cells (BT6 and BT18), the MCF-7, BT-20 and MDA-MB-435 tumor lines, and NKG2D–DAP10 transfected (MCF-7–TF), NKG2D depleted (MCF-7–TF–KO, MCF-7–NKG2D RNAi), and mock-transfected (MCF-7 mock) and scrRNAi-transduced (MCF-7–scrRNAi) control derivatives of MCF-7. Red lines in histograms represent isotype control stainings. (**A**) Minimal surface NKG2D on parental and mock-transfected MCF-7 cells. (**B**) Minimal surface NKG2D on BT-20 and MDA-MB-453 cells. (**C**) Representative examples of surface NKG2D on *ex* *vivo* breast cancer cells gated for EpCAM^+^CD45^–^. (**D**) RT PCR of mRNA for NKG2D, DAP10, and control GAPDH from MCF-7–TF and MCF-7–TF–KO cells, and from CD45^–^EpCAM^+^ breast cancer cells sorted from seven surgical breast cancer (BT) specimens (**E**). NKG2D immunoprecipitation (IP) and immunoblotting (IB) for NKG2D and DAP10 using cell lysates of MCF-7–TF and MCF-7–TF–KO cells, and of sorted CD45^–^EpCAM^+^ breast cancer cells corresponding to five of the BT samples shown in (**D**). (**F**) Profiles of MCF-7–TF and MCF-7–TF–KO cells. (**G**) Profiles of MCF-7–NKG2D RNAi and MCF-7–scrRNAi cells. (**H**) Immunoblot detection of phosphorylated AKT (S473) after anti-NKG2D mAb 1D11 crosslinking in sorted CD45^–^EpCAM^+^ breast cancer cells as compared to negative control conditions. Insulin was added for control activation, DMSO for solvent control. LY294002 is an inhibitor of PI3K.
(TIF)

Figure S2 **Signaling proficiency of the MDAH-2774 and MCF-10AT tumor lines with ectopic expression of NKG2D–DAP10.** (**A**) Flow cytometry of the MDAH-2774–TF and MCF-10AT–TF cells with constitutive and Dox-inducible surface NKG2D, respectively. (**B**) Immunoprecipitation (IP) and immunoblot (IB) of NKG2D and the associated DAP10. (**C**) Immunoblot detection of phosphorylated AKT (S473) and ERK (T202/Y204) after anti-NKG2D mAb 1D11 crosslinking in MDAH-2774–TF and Dox-induced MCF-10AT–TF cells as compared to negative control conditions. LY294002 and U0126 are inhibitors of PI3K and MEK/ERK, respectively. EGF was added for control activation, DMSO for solvent control.
(TIF)

Figure S3 **Induction of EMT-associated changes by conditionally expressed NKG2D–DAP10 in virally transduced MCF-10AT–TF, and transfected SUM149PT–TF, MDAH-2774–TF, and A375–TF cells.** (**A**) Phase contrast microscopy shows epithelial to mesenchymal transdifferentiation of Dox-induced MCF-10AT–TF cells versus negative controls. By immunofluorescence microscopy, induced MCF-10AT–TF cells display diminished E-cadherin, and induced N-cadherin and vimentin. (**B**) Confirmatory immunoblot (left panel) and RT-PCR (right panel) data including an expanded set of diagnostic markers. (**C**) RT-PCR transcription factor profiles from Dox-induced MCF-10AT–TF versus control lines. (**D**) Profiling of SUM149PT–TF, MDAH-2774–TF, A375–TF, and untransfected/mock controls for diagnostic EMT markers by immunoblot (top panel) and RT-PCR (bottom panel). Melanoma A375 cells are negative for E-cadherin (17). (**E**) Flow cytometry of induced MCF-10AT–TF cells and negative controls grown to low or high confluence for E-cadherin and N-cadherin. Numbers in quadrants indicate cell proportions in percent. Note that this detection is more sensitive then the procedure used in (**A**). (**F**) Graphic display of *in* *vitro* migration and invasion data. Bars represent mean cell numbers derived from three independent experiments with each four microscopic field counts. Asterisks denote *p*<0.005.
(TIF)

File S1 **Supporting Materials and Methods S1–S3.**
(DOC)

Table S1 **Histopathological data for breast tumor specimens and proportional subsets of NKG2D positive cells.** Abbreviations: BT, breast tumor; ER, estrogen receptor; PR, progesteron receptor; HER2, human epidermal growth factor receptor 2; E, E-cadherin; N, N-cadherin; IDC, invasive ductal carcinoma; Ind, indeterminate. ?, unknown.
(DOC)
