# Supplementary figures and images for: Autonomous Stimulation of Cancer Cell Plasticity by the Human NKG2D Lymphocyte Receptor Coexpressed with Its Ligands on Cancer Cells
Source: PLoS One. 2014 Oct 7;9(10):e108942. doi: 10.1371/journal.pone.0108942 (PMC4188595; doi:10.1371/journal.pone.0108942)

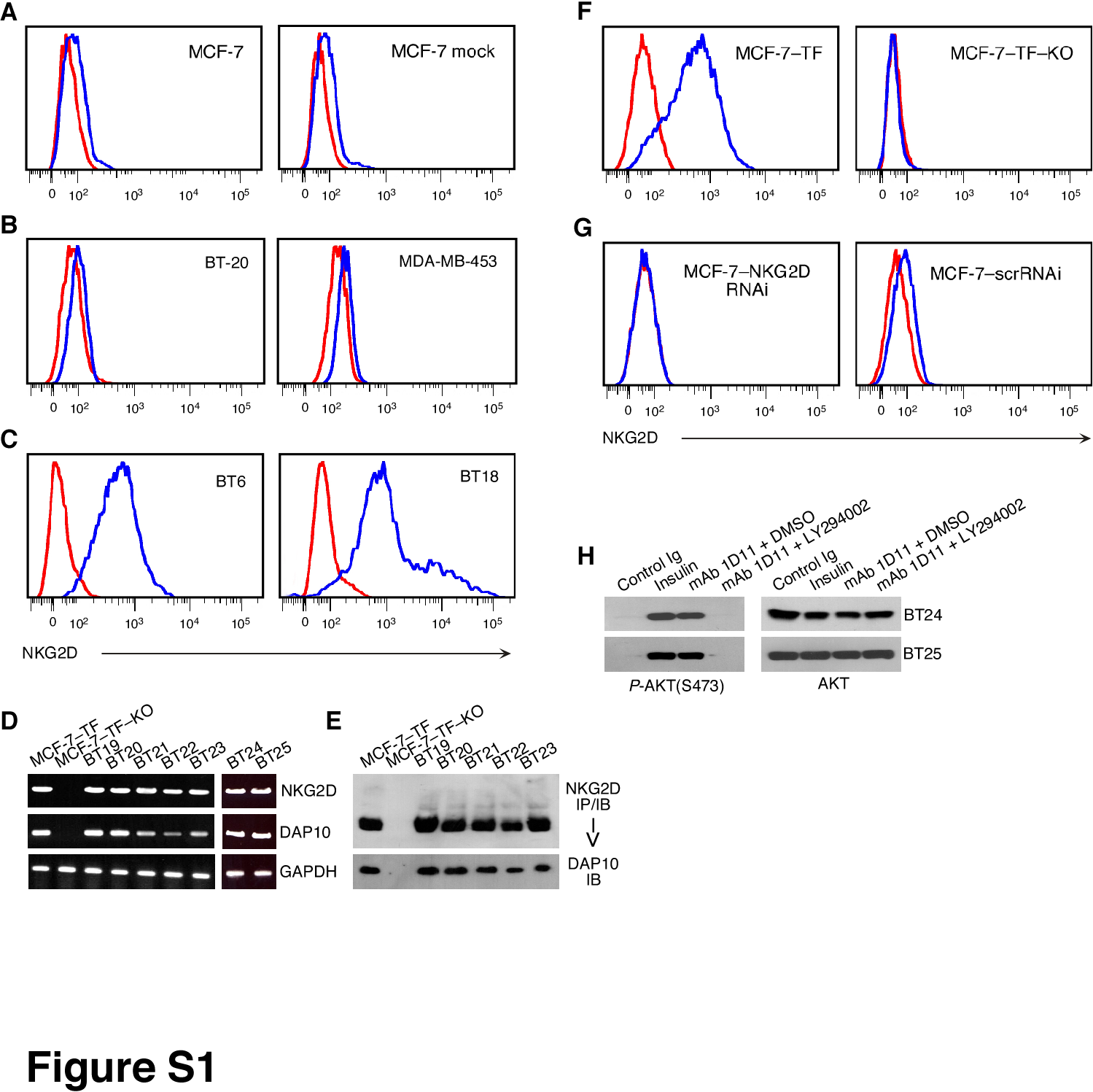

Supplement: Figure S1 — NKG2D–DAP10 in breast tumor lines and ex vivo breast cancer cells. (A–C, F, G) Flow cytometry profiles of surface NKG2D (blue lines) on ex vivo breast cancer cells (BT6 and BT18), the MCF-7, BT-20 and MDA-MB-435 tumor lines, and NKG2D–DAP10 transfected (MCF-7–TF), NKG2D depleted (MCF-7–TF–KO, MCF-7–NKG2D RNAi), and mock-transfected (MCF-7 mock) and scrRNAi-transduced (MCF-7–scrRNAi) control derivatives of MCF-7. Red lines in histograms represent isotype control stainings. (A) Minimal surface NKG2D on parental and mock-transfected MCF-7 cells. (B) Minimal surface NKG2D on BT-20 and MDA-MB-453 cells. (C) Representative examples of surface NKG2D on ex vivo breast cancer cells gated for EpCAM+CD45–. (D) RT PCR of mRNA for NKG2D, DAP10, and control GAPDH from MCF-7–TF and MCF-7–TF–KO cells, and from CD45–EpCAM+ breast cancer cells sorted from seven surgical breast cancer (BT) specimens (E). NKG2D immunoprecipitation (IP) and immunoblotting (IB) for NKG2D and DAP10 using cell lysates of MCF-7–TF and MCF-7–TF–KO cells, and of sorted CD45–EpCAM+ breast cancer cells corresponding to five of the BT samples shown in (D). (F) Profiles of MCF-7–TF and MCF-7–TF–KO cells. (G) Profiles of MCF-7–NKG2D RNAi and MCF-7–scrRNAi cells. (H) Immunoblot detection of phosphorylated AKT (S473) after anti-NKG2D mAb 1D11 crosslinking in sorted CD45–EpCAM+ breast cancer cells as compared to negative control conditions. Insulin was added for control activation, DMSO for solvent control. LY294002 is an inhibitor of PI3K. (TIF) [file pone.0108942.s001.tif]

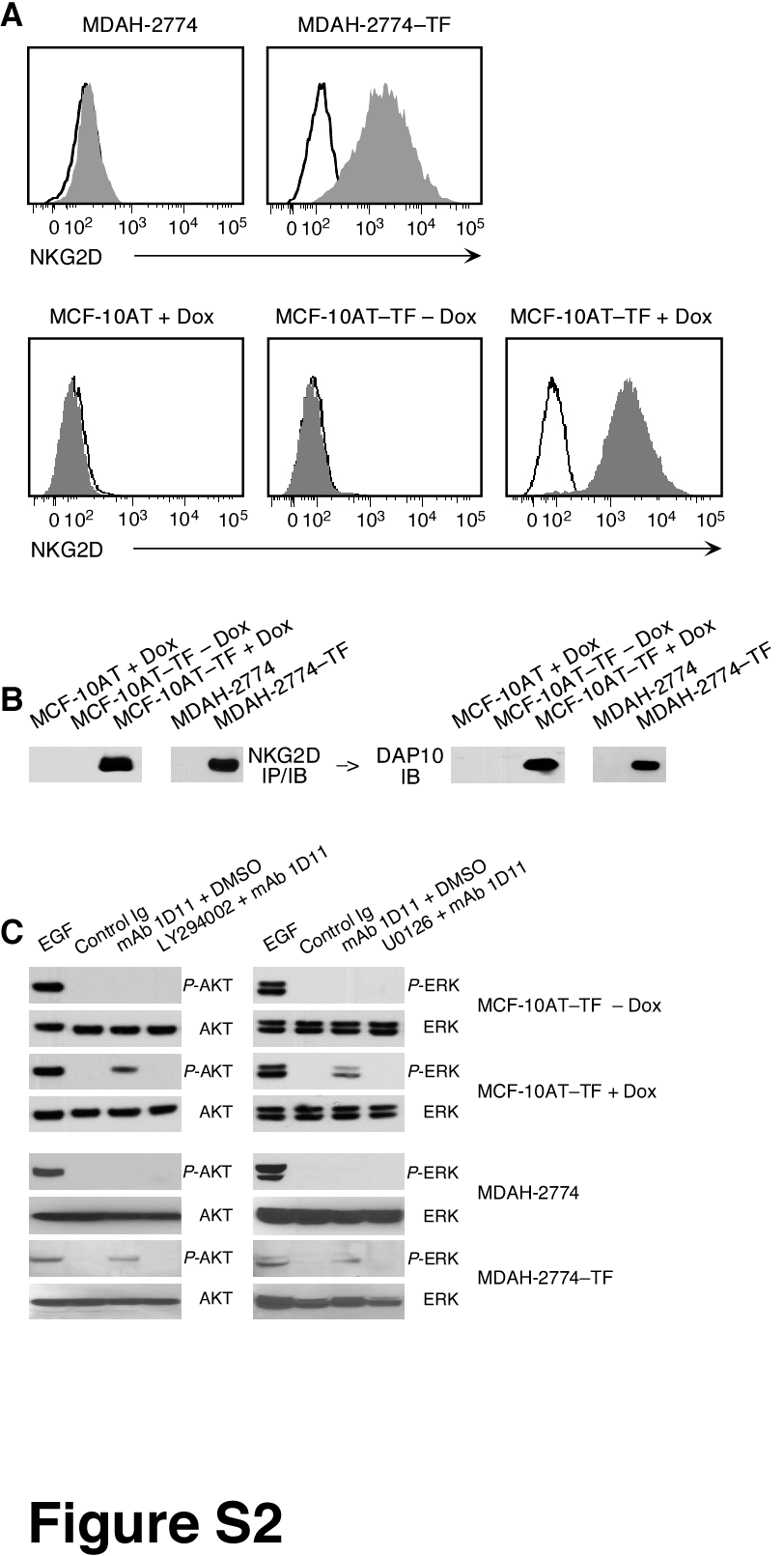

Supplement: Figure S2 — Signaling proficiency of the MDAH-2774 and MCF-10AT tumor lines with ectopic expression of NKG2D–DAP10. (A) Flow cytometry of the MDAH-2774–TF and MCF-10AT–TF cells with constitutive and Dox-inducible surface NKG2D, respectively. (B) Immunoprecipitation (IP) and immunoblot (IB) of NKG2D and the associated DAP10. (C) Immunoblot detection of phosphorylated AKT (S473) and ERK (T202/Y204) after anti-NKG2D mAb 1D11 crosslinking in MDAH-2774–TF and Dox-induced MCF-10AT–TF cells as compared to negative control conditions. LY294002 and U0126 are inhibitors of PI3K and MEK/ERK, respectively. EGF was added for control activation, DMSO for solvent control. (TIF) [file pone.0108942.s002.tif]

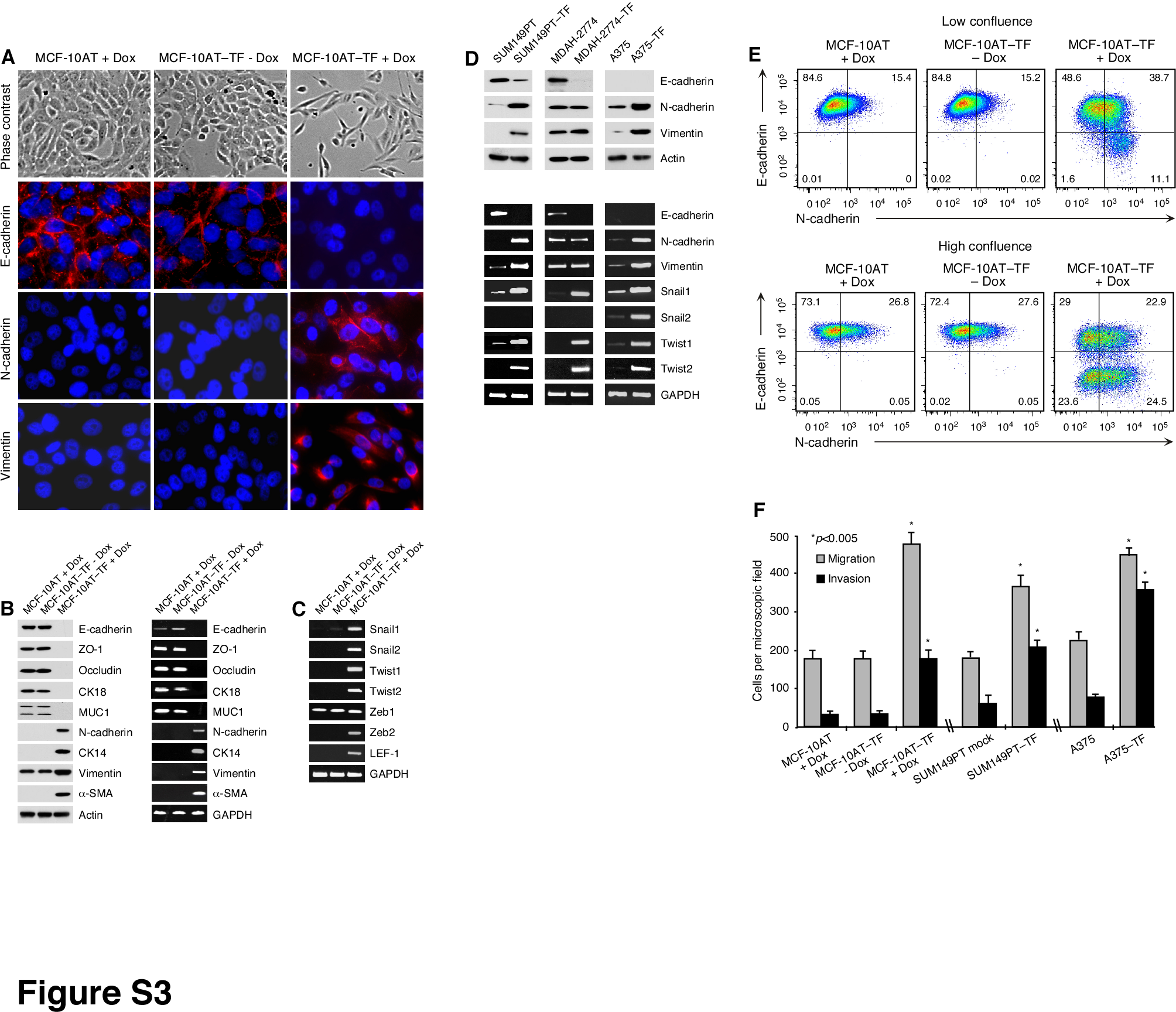

Supplement: Figure S3 — Induction of EMT-associated changes by conditionally expressed NKG2D–DAP10 in virally transduced MCF-10AT–TF, and transfected SUM149PT–TF, MDAH-2774–TF, and A375–TF cells. (A) Phase contrast microscopy shows epithelial to mesenchymal transdifferentiation of Dox-induced MCF-10AT–TF cells versus negative controls. By immunofluorescence microscopy, induced MCF-10AT–TF cells display diminished E-cadherin, and induced N-cadherin and vimentin. (B) Confirmatory immunoblot (left panel) and RT-PCR (right panel) data including an expanded set of diagnostic markers. (C) RT-PCR transcription factor profiles from Dox-induced MCF-10AT–TF versus control lines. (D) Profiling of SUM149PT–TF, MDAH-2774–TF, A375–TF, and untransfected/mock controls for diagnostic EMT markers by immunoblot (top panel) and RT-PCR (bottom panel). Melanoma A375 cells are negative for E-cadherin (17). (E) Flow cytometry of induced MCF-10AT–TF cells and negative controls grown to low or high confluence for E-cadherin and N-cadherin. Numbers in quadrants indicate cell proportions in percent. Note that this detection is more sensitive then the procedure used in (A). (F) Graphic display of in vitro migration and invasion data. Bars represent mean cell numbers derived from three independent experiments with each four microscopic field counts. Asterisks denote p<0.005. (TIF) [file pone.0108942.s003.tif]
