# Supplementary material for: Autonomous Stimulation of Cancer Cell Plasticity by the Human NKG2D Lymphocyte Receptor Coexpressed with Its Ligands on Cancer Cells
Source: PLoS One. 2014 Oct 7;9(10):e108942. doi: 10.1371/journal.pone.0108942 (PMC4188595; doi:10.1371/journal.pone.0108942)
Supplement: File S1 — Supporting Materials and Methods S1–S3. (DOC) [file pone.0108942.s004.doc]

**Supporting Materials and Methods**

**Materials and Methods S1. NKG2D–DAP10 expression, siRNA transduction, and RT-PCR.** Dox-inducible expression of NKG2D (*KLRK1*; GenBank accession number X54870), and DAP10 (*HCST*; GenBank accession number AF072844) and its N87Q* and M88Q* mutants, was directed by insertion of RT-PCR amplicons flanked by *Bam* HI and *Sma* I sites (underlined) into lentiviral pLVCT-tTR-KRAB constructs (plasmid 11643, Addgene). The primers used (forward and reverse, 5’–3’) were CGCGGATCCACCGGTCGCCACCATGGGGTGGATTCGTG

GTCGGAGG and TCCCCCGGGTTACACAGTCCTTTGCATGCAG (NKG2D), and CGCGGATC

CACCGGTCGCCACCATGGCCTTACCAGTGACCGCC and TCCCCCGGGTCAGCCCCTGCC

TGGCATGTTG (DAP10). For virus production, 293T cells on poly-L-lysine-coated plates were transfected with expression constructs together with envelope helper (pMD2.G) and gag/pol helper (pCMVR8.74) plasmids using calcium phosphate. Virus in culture supernatants was concentrated by centrifugation, titered and used for transduction aided by protamine sulfate (MP Biomedicals). MCF10AT cells were co-infected with lentiviral NKG2D and DAP10 constructs in the presence of Dox (1 μg/ml, Clontech) and sorted for surface NKG2D positive cells.

Oligonucleotide primers (forward and reverse, 5’–3’) for RT-PCR of EMT-associated transcription factors and cellular markers were ACCTTCCAGCAGCCCTACGACC and GTGTG

GCTTCGGATGTGCATC (Snail1), ATTCGGACCCACACATTACCTTG and TGGAGAAGGTTT

TGGAGCAGTTT (Snail2), CAGGGCCGGAGACCTAGATGTCATTG and GCACGACCTCTTG

AGAATGCATGCATG (Twist1), GCCGCCAGGTACATAGACTT and CCCCAAACATAAGACC

CAGA (Twist2), TTCAGCATCACCAGGCAGTC and GAGTGGAGGAGGCTGAGTAG (Zeb1), CGCTTGACATCACTGAAGGA and CTTGCCACACTCTGTGCATT (Zeb2), GAACGAGTCTGA

AATCATCC and GTAGGGATATCAGGAGCTGG (LEF-1), CGGGAATGCAGTTGAGGATC and

AGGATGGTGTAAGCGATGGC (E-cadherin), CACTGCTCAGGACCCAGAT and TAAGCCGAG

TGATGGTCC (N-cadherin), AGGAAATGGCTCGTCACCTTCGTGAATA and GGAGTGTCGGT

TGTTAAGAACTAGAGCT (vimentin), GGAGATGATTGGCAGCGTGGAG and AGAACTGGGA

GGAGGAGAGGTG (CK14), TGAGACGTACAGTCCAGTCCTT and GCTCCATCTGTAGGGC

GTAG (CK18), GGTCAGTGTGGAGGTGGATT and TCAGTAACCTCGGACCTGCT (CK19), C

TGTTCCAGCCATCCTTCAT and CCGTGATCTCCTTCTGCATT (α-SMA), TGCATGTTCGACC

AATGC and AAGCCACTTCCTCCATAAGG (occludin), CATAGAATAGACTCCCCTGG and GC

TTGAGGACTCGTATCTGT (ZO-1), GTGCCCCCTAGCAGTACCG and GACGTGCCCCTACAA

GTTGG (MUC1), AGACCTTTGGGCTGCCTTAT and TAGCCTCCCTCACTCCAAGA (Sox9), and AGCCACATCGCTCAGACACC and GATACCCTTTTGGCTCCCC (GAPDH). PCR employed 30 cycles at 94°C (15 s), 62°C (30 s), and 72°C (60 s) using the SuperScript III One-Step RT-PCR System with Platinum Taq (Invitrogen). Two-step real-time PCR of Sox9 employed SuperScript III First-Strand Synthesis SuperMix and Platinum SYBR Green qPCR SuperMix-UDG (Invitrogen) with primers CATGAGCGAGGTGCACTCC and TCGCTTCAGGTC

AGCCTTG. Snail2 was amplified in 40 cycles in the experiment with MCF-7–NKG2D RNAi and negative control cells. The DAP10 N87Q* and M88Q* variants were generated using a QuikChange Site-Directed Mutagenesis Kit (Stratagene).

**Materials and Methods S2. Sample preparation for gene expression microarrays.** RNA samples of MCF-7–mock and MCF-7–TF cells were extracted using RNeasy Plus Mini Kit (Qiagen) and quality controlled using an Agilent 2100 Bioanalyzer (Agilent Technologies). RNA samples were converted to cDNA and biotin-labeled for microarray analysis using Ambion’s Illumina TotalPrep RNA Amplification kit (Life Technologies).

**Materials and Methods S3. Tumor cell invasion/migration assays.** Upper compartments of BD BioCoat Matrigel Invasion Chambers (Discovery Labware) were seeded with mock-transfected MCF-7, MCF-7–TF, or MCF-7–TF–NKG2D RNAi cells (each 5 x 10^4^ cells, harvested with EDTA in PBS and washed in RPMI 1640 medium). Bottom compartments contained RPMI 1640 plus FBS (10%) as chemoattractant. After incubation for five days, non-invading cells were removed from the upper membrane surface with a cotton swab and invading cells that had traversed the matrigel to the lower membrane surface stained with Diff-Quik stain (Fisher) and counted in four randomly selected microscopic fields. Experiments were performed at least three times for each experimental condition.
