# Supplementary material for: Autonomous Stimulation of Cancer Cell Plasticity by the Human NKG2D Lymphocyte Receptor Coexpressed with Its Ligands on Cancer Cells
Source: PLoS One. 2014 Oct 7;9(10):e108942. doi: 10.1371/journal.pone.0108942 (PMC4188595; doi:10.1371/journal.pone.0108942)
Supplement: Table S1 — Histopathological data for breast tumor specimens and proportional subsets of NKG2D positive cells. Abbreviations: BT, breast tumor; ER, estrogen receptor; PR, progesteron receptor; HER2, human epidermal growth factor receptor 2; E, E-cadherin; N, N-cadherin; IDC, invasive ductal carcinoma; Ind, indeterminate. ?, unknown. (DOC) [file pone.0108942.s005.doc]

**Table S1.**

Histopathological data for breast tumor specimens and proportional subsets of NKG2D positive cells

| **BT** | **Primary Diagnosis** | **Grade** | **ER**  **status** | **PR**  **status** | **HER2 status** | **% NKG2D^+^ cells among CD45^–^EpCAM^high^ cells** | **% NKG2D^+^ cells among CD45^–^**  **EpCAM^low^ cells** | **% NKG2D^+^ cells among CD45^–^ EpCAM^+^ E^+^/N^+^ and E^–^/N^+^ cells** |
| --- | --- | --- | --- | --- | --- | --- | --- | --- |
|  |  |
|  |  |  |  |  |  |
| 1 | IDC | 3 | Pos | Pos | Ind | 0.444 | 1.914 | 40.714 |
| 2 | IDC | 2 | Pos | Neg | Neg | 5.15 | 17.83 | 88.43 |
| 3 | IDC | 3 | Pos | Pos | Neg | 13.3 | 59.3 | 94.5 |
| 4 | IDC | 2 | Pos | Pos | Neg | 3.693 | 0.3 | 8.47 |
| 5 | IDC | 2 | Pos | Pos | Neg | 4.86 | 5.97 | 31.46 |
| 6 | IDC | 3 | Pos | Pos | Neg | 10.39 | 0.3 | 5.23 |
| 7 | IDC | 2 | Pos | Pos | Ind | 2.034 | 0.282 | 33.814 |
| 8 | IDC | 3 | Pos | Neg | Pos | 11.45 | none | 53.455 |
| 9 | IDC | 3 | Pos | Pos | Ind | 1.75 | 0.31 | 46.77 |
| 10 | IDC | 3 | Pos | Pos | Neg | 27.77 | 6.53 | 70 |
| 11 | IDC | 3 | Neg | Neg | Neg | 26.2 | 9.48 | 53.1 |
| 12 | IDC | 3 | Neg | Neg | Neg | 9.67 | 36 | 31.5 |
| 13 | IDC | 3 | Pos | Pos | Neg |  | | |
| 14 | IDC | 3 | Pos | Pos | Pos |  | | |
| 15 | IDC | 2 to 3 | Pos | Pos | Neg |  | | |
| 16 | IDC | 2 to 3 | Pos | Pos | Ind |  | | |
| 17 | IDC | 2 | Pos | Pos | Pos |  | | |
| 18 | IDC | 3 | Pos | Pos | Neg |  | | |
| 19 | IDC | 2 to 3 | Pos | Pos | Ind |  | | |
| 20 | IDC | 2 | ? | ? | ? |  | | |
| 21 | IDC | 2 to 3 | Pos | Pos | Ind |  | | |
| 22 | IDC | ? | ? | ? | ? |  | | |
| 23 | IDC | 2 | ? | ? | ? |  | | |
| 24 | IDC | ? | ? | ? | ? |  | | |
| 25 | IDC | 2 to 3 | Pos | Pos | Ind |  | | |

Abbreviations: BT, breast tumor; ER, estrogen receptor; PR, progesteron receptor; HER2, human epidermal growth factor receptor 2; E, E-cadherin; N, N-cadherin; IDC, invasive ductal carcinoma; Ind, indeterminate. ?, unknown.
